# *KAT2B* Gene Polymorphisms Are Associated with Body Measure Traits in Four Chinese Cattle Breeds

**DOI:** 10.3390/ani12151954

**Published:** 2022-08-01

**Authors:** Xiaoding Lin, Bo Li, Yuhan Chen, Hong Chen, Mei Liu

**Affiliations:** 1 College of Animal Science and Technology, Hunan Agricultural University, Changsha 410128, China; xiaodinglinn@163.com (X.L.); chenyuhan180@163.com (Y.C.); 2 Shaanxi Key Laboratory of Molecular Biology for Agriculture, College of Animal Science and Technology, Northwest A&F University, No.3 Taicheng Road, Xianyang 712100, China; libozm@126.com

**Keywords:** KAT2B, SNP, cattle, body measure traits, association analysis

## Abstract

**Simple Summary:**

Genetic improvement is one of the most important keys to overcoming the shortcomings of beef production. Identifying molecular markers related to growth characteristics and meat quality is significant in improving beef cattle breeds. Studies have shown that *KAT2B*, a transcriptional co-activator regulating the acetylation modification of histones, may be involved in the development and metabolism of muscle and adipose. However, there are no reports on investigating *KAT2B* genetic variation in Chinese native cattle. Firstly, this manuscript reports the initial bioinformatics analysis of KAT2B, finding that KAT2B protein is highly conserved among ruminants. The *KAT2B* gene expression profile in Qinchuan cattle was characterized, showing the spatiotemporal specificity of *KAT2B* gene expression in tissues such as adipose and liver during fetal and adult periods. Then, the investigation of *KAT2B* gene polymorphisms was carried out. Three SNPs of the *KAT2B* gene were identified and were found to be correlated with multiple body measurements in Fu, Qinchuan, Yak, and Chaidam cattle. These findings suggest that these three SNPs of *KAT2B* can serve as the molecular markers to select individuals for beef cattle breed improvement.

**Abstract:**

Identifying molecular markers related to growth characteristics or meat quality is significant for improving beef cattle breeds. K(lysine) acetyltransferase 2B (*KAT2B*) is a transcriptional co-activator regulating the acetylation modification of histones, which may be involved in the development and metabolism of muscle and adipose. However, investigations of *KAT2B* genetic variations in Chinese native cattle are still limited. This study aimed to identify crucial single nucleotide polymorphisms (SNPs) influencing the body measurements of Chinese native cattle. Biological evolution and conservation analysis showed that KAT2B was highly conserved among the ruminants. By qPCR assay, *KAT2B* gene expression was found to be spatiotemporally specific in bovine tissues such as adipose and liver. By the RFLP-PCR method, three SNPs of *KAT2B* (g.T61908C, g.T62131C, and g.C73406T) were identified in 827 individuals of four Chinese cattle breeds, including Qinchuan (*n* = 658), Fu (*n* = 52), Yak (*n* = 48), and Chaidam (*n* = 69) cattle. Association analysis between these *KAT2B* polymorphisms and the body measurements of Chinese native cattle revealed significant observations. The genetic effects of g.T61908C, g.T62131C, and g.C73406T on the associated phenotypes were illustrated in each breed. In Qinchuan cattle, g.T62131C was significantly associated with better body height, chest width, hip width, and withers height, for which TC and/or TT were the advantageous genotype. In Fu cattle, TT genotype of g.T61908C was associated to better body length, while individuals with TT or CC of g.T62131C showed higher circumference of cannon bone than those with TC genotype. In Yak, individuals with TT genotype of g.C73406T had heavier body weight. In Chaidam cattle, TC genotype of g.C73406T was associated to superior body weight, while CC genotype of g.C73406T was associated to superior chest girth and circumference of cannon bone. These findings suggest that *KAT2B* gene polymorphisms can be used as the molecular markers for the early molecular marker-assisted selection in beef cattle breeding programs.

## 1. Introduction

China has a wealth of local cattle breeds, but most of them have obvious shortcomings in beef production, such as small body size, low growth rate, and low meat yield [1], which limit their use in industrial production and economic value. Most of the body measure traits are heritable to varying levels and have been widely reported in cattle. Among the factors contributing to the economic traits of livestock, genetic improvement is very crucial because genetic information is permanent and cumulative when inherited by subsequent generations [2]. DNA is inherited through generations by vertical transmission from parents to offspring and from ancestor to descendant species. Therefore, identifying the functional genes and their DNA genetic variation is very import for the beef cattle industry.

Molecular marker-assisted selection using SNPs is an effective means to improve livestock in modern animal breeding. It selects a phenotype based on a genotype tightly linked to the phenotype [3]. Early selective breeding based on genomics can increase the rate of genetic modification and reduces the cost of progeny testing [4,5,6]. Therefore, the selection of potential sites that affect the beef quality and yield has attracted widespread attention in cattle breeding [7]. Many SNPs and genes have been reported to be useful for breeding purposes in cattle [7]. However, only a few genes and QTLs (quantitative trait loci) have been determined for body measurements in Chinese cattle breeds [8]. An urgent need is to find more causal loci of cattle growth and beef characteristics, to facilitate the genetic improvement of Chinese native cattle breeds.

K(lysine) acetyltransferase 2B (*KAT2B*), also known as PCAF (p300/CBP-associated factor), is a transcriptional co-activator. It can bind histones and other proteins to form large protein complexes to regulate histone acetylation modification [9]. Studies have shown that KAT2B could dramatically reduce acetylation of H3K9 in mammalian cells [9,10]; and have functions in DNA replication and repair [11], cell cycle and death, actin-mediated cell contraction, protein transport, and regulation of centrosome duplication [12]. Moisa, S. J., et al. [13] found that the expression of *KAT2B* in the longissimus muscle had a time × feed treatment interaction during the development of Angus and Angus × Simmental steers, suggesting that *KAT2B* might be involved in cattle’s nutritional metabolism and growth. Furthermore, in our previous study, *KAT2B* was found to be significantly differentially expressed between the fetal and adult Qinchuan cattle, implying that it may be a candidate gene involved in bovine fat deposition [14]. However, up to now, there are no reports on investigating *KAT2B* genetic variation in Chinese native cattle. Thus, it is necessary to detect whether genetic variation in the *KAT2B* gene can affect the body measurements of Chinese native cattle.

This study first reports the initial bioinformatics analysis for biological evolution and conservation of KAT2B in different species. Then, the *KAT2B* gene expression profile was investigated in Qinchuan cattle. The detection of *KAT2B* gene polymorphisms was carried out in four Chinese native cattle breeds (Fu, Qinchuan, Yak, and Chaidam cattle). The association analysis between the candidate SNPs of the *KAT2B* gene and body measurements of those Chinese native cattle was performed in this study. Our findings provide novel molecular markers, the SNPs of the *KAT2B* gene, for Chinese beef cattle breeding.

## 2. Materials and Methods

### 2.1. Bioinformatics Analysis

The amino acid sequences of the KAT2B protein were downloaded from NCBI (https://www.ncbi.nlm.nih.gov/protein, accessed on 29 January 2022). Sequence alignment and phylogenetic tree of KAT2B protein forming were performed among different species, including *Bos taurus* (XP_024853358.1), *Bos idicus* (XP_019822500.1), *Bos mutus* (ELR48408.1), *Capra hircus* (XP_017908727.1), *Ovis aries* (XP_027817893.1), *Bubalus bubalis* (XP_025150061.1), *Sus scrofa* (XP_020927025.1), *Homo sapiens* (NP_003875.3), *Rattus rattus* (XP_032756718.1), *Mus musculus* (NP_001177775.1), and *Gallus gallus* (XP_040519860.1) by MEGA X 10.2.6 (Philadelphia, PA, USA) (https://www.megasoftware.net/, accessed on 21 February 2022) [15]. Aligning multiple sequences and forming a phylogenetic tree were performed by MUSCLE sequencing alignment and neighbor-joining (NJ) method, respectively [15]. To reveal the structural characteristics and functions of *KAT2B* proteins in the above 11 species, we investigated the motifs through the MEME suite (https://meme-suite.org/, accessed on 22 Febuary 2022) [16]. The conserved domains were analyzed through CDD from NCBI (https://www.ncbi.nlm.nih.gov/cdd/, accessed on 4 March 2022) [17].

### 2.2. Animals, DNA Sampling, and Phenotype Data Collection

According to the relevant local laws and policies on animal welfare and institutional guidelines of the Institutional Animal Protection and Use Committee of Northwest A&F University (protocol number: NWAFAC1008), the following animal experiments in this study were approved and conducted.

A total of 827 female cattle blood samples were collected from four Chinese local cattle breeds, including Qinchuan (*n* = 658), Fu (*n* = 52), Yak (*n* = 48) and Chaidam (*n* = 69). Among them, Qinchuan cattle, one of the most representative Chinese local cattle breeds, ranks first among the yellow cattle in China. Chaidam cattle is the local yellow breed in Qinhai province. Fu is the Angus–Yak–Chaidam crossbreed. For individuals in each population, the animals were non-relatives for at least three generations. All the individuals were two years old. According to standard procedures, genomic DNA was isolated from blood samples and stored at −80 °C.

We measured phenotypic traits in cattle according to the method provided in Molecular Cloning—a laboratory manual [18]—including body mass (BM), body height (BH), body length (BL), chest width (CW), the circumference of the cannon bone (CCB), hip width (HIW), chest girth (CG), and waist height (WH). The analysis of these phenotypic traits’ distribution in each breed was performed by GraphPad Prism version 8.0.0 software (San Diego, CA, USA) (Figure S1).

### 2.3. RNA Extraction, cDNA Synthesis, and Real-Time Quantitative PCR (qPCR)

Six female Qinchuan cattle individuals, including three fetal bovines and three female adult bovines, were randomly selected for slaughter. Seven tissues, including spleen, lung, kidney, heart, longissimus *dorsi*, liver, and the subcutaneous fat of the back, were collected and frozen in liquid nitrogen immediately after slaughter and then stored in a −80 °C refrigerator for further analysis. The RNA was extracted using Trizol. The cDNA was synthesized with a PrimeScript^TM^ RT reagent Kit with gDNA Eraser (Takara, Kusatsu, Japan) based on the recommended procedure.

Using the SYBR Green series (Takara), real-time quantitative PCR (qRT-PCR) was performed to detect the gene expression. β-actin was used as a reference gene for normalization [19]. The primer P (Table 1) for qRT-PCR was designed based on the *KAT2B* gene mRNA sequence (XM_019966941.1) with Primer 5.0 software (Sangon Biotech (Shanghai) Co., Ltd., China). The PCR was run as the previous protocol [1]: in brief, 95 °C for 5 min, 34 cycles, 95 °C for 30 s, 60 °C for 30 s, and 72 °C for 30 s. The qRT-PCR was conducted in triplicate for each sample.

### 2.4. Detection of KAT2B SNPs by DNA Pool Sequencing

To explore the allele variation of the bovine *KAT2B* gene, a total of 17 primer pairs were designed to cover the exons and the adjacent introns (Table S1, GenBank accession number: AC_000158.1). Forty DNA samples were randomly selected from each cattle breed, to construct for DNA pools of four breeds [19]. PCR reactions were carried out using a Touchdown PCR System Thermal Cycler Dice (TaKaRa, Dalian, China) and a PCR protocol which was described in a previous study [20]. DNA pool sequencing for the PCR productions using a Sanger sequencing strategy was applied to screen the genetic variations. The sequences were imported into the BioXM software (version 2.6) to search for SNPs.

### 2.5. Genotyping for KAT2B SNPs by PCR–RFLP

Based on the results of *KAT2B* SNPs detection, the polymerase chain reaction-restriction fragment length polymorphism (PCR-RFLP) technique was used for genotyping of the candidate SNPs of *KAT2B* gene. Three pairs of primers were designed by Primer 5.0 software (Sangon Biotech (Shanghai) Co., Ltd., Shanghai China), targeting the fragments of g.C61908T (P1), g.T62131C (P2), and g.C73406T (P3) polymorphic sites of the *KAT2B* gene (Table 1). The PCR reactions were carried out in a 25 μL volume which contained 1 μL cattle genomic DNA (50 ng/μL), 0.5 μL each primer (10 μmol/L), 12.5 μL 2 × Taq PCR MasterMix (Taq DNA polymerase, Mg^2+^, dNTPs, et al.), and ddH_2_O. The PCR tests were carried out with the PCR System Thermal Cycler Dice (TaKaRa, Dalian, China), with the following amplification protocol: 94 °C for 5 min; followed by 35 cycles of 94 °C for 30 s, annealing at selected temperatures (Table 1) for 30 s, and extension at 72 °C for a 30 s; and a final extension at 72 °C for 10 min [20].

Then, to genotype the three SNPs of *KAT2B*, the PCR products of g.C61908T, g.T62131C, and g.C73406T were digested, respectively, with restriction endonucleases *Hind* III, *Apa* I, and *Apa* I (TaKaRa, Tokyo, Japan), with the method described in our previous study [20]. The digested products were detected by electrophoresis in 3.0% agarose gel stained with 200 ng/mL ethidium bromide at a constant voltage (120 V) for 40 min. The patterns of DNA bands were observed and photographed with the Bio-rad Gel Doc 2000 Gel Imaging system.

### 2.6. Statistical Analysis

The relative expression levels of the *KAT2B* gene were calculated via 2^−∆∆Ct^ method [21]. The mRNA expression levels of *KAT2B* in tissues were analyzed through GraphPad Prism 6 software.

The allelic and genotypic frequencies of all three SNPs were calculated by Haploview software [22]. He (gene heterozygosity), Ne (effective allele numbers), and *PIC* (polymorphism information content) were calculated according to Nei’s methods, respectively [23,24]. The Hardy–Weinberg equilibrium (HWE) was calculated using the chi-square test [25].

The association analysis between genotypes and growth traits was performed with a general linear model procedure, and they were compared by Duncan’s Multiple Range Test (SPSS V19.0, Inc., Chicago, IL, USA) software. The equation is as follows: $Y_{ijk}=\mu +G_{i}+F_{j}+B_{k}+e_{ijk}$. *Y_ijk_* represents the phenotypic observations; *μ* represents the average values; *G_i_* is the fixed effect of genotype; $F_{j}$ represents the fixed effect of farm; $B_{k}$ is the fixed effect of cattle breeds; *e_ijk_* is the residual effect. All values are presented as the mean ± SE. *p* values < 0.05 were considered as statistically significant.

## 3. Results

### 3.1. Biological Evolution and Conservation Analysis of KAT2B

**Figure 1:**
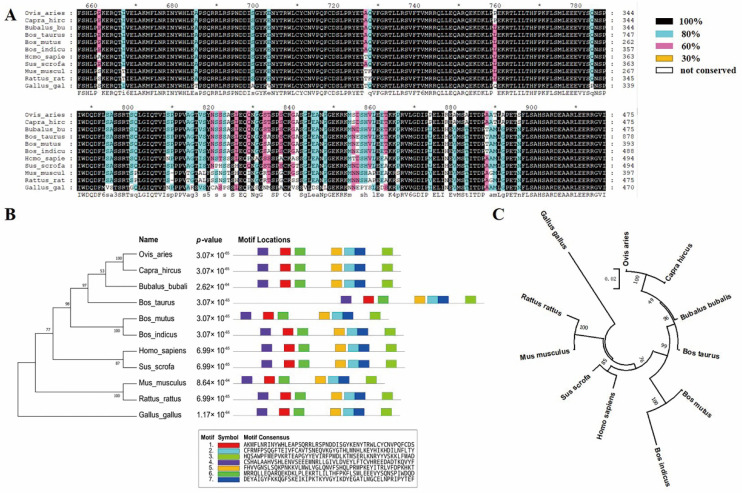
Biological evolution and conservation of KAT2B. (**A**) Multiple sequence alignment of KAT2B for the 11 species. The degree of similarity is delineated using different background shading: black−100%, blue−80%, pink−60%, yellow−30%, and white−not conserved. (**B**) Phylogenetic tree (Left) and motif structural analysis (Right) for the 11 species. Seven significant motifs were identified. The length of the color block shows the position, strength, and significance of a particular motif site. The motif length is proportional to the negative logarithm of the *p*-value of the motif site, truncated at the height for a *p*-value of 1 × 10^−10^. These were are given by motif analysis performed through the MEME suite system. (**C**) Phylogenetic tree for the 11 species.

The multiple sequence alignment of KAT2B proteins was performed for eleven mammalian or domestic animals, including cattle (*Bos taurus*), zebu cattle (*Bos indicus*), yaks (*Bos mutus*), buffalo (*Bubalus bubalis*), goats (*Capra hircus*), sheep (*Ovis aries*), pigs (*Sus scrofa*), humans (*Homo sapiens*), mice (*Mus musculus*), rats (*Rattus rattus*), and chickens (*Gallus gallus*). The results show that KAT2B protein structure was highly conserved among the ruminants, such as cattle, zebu cattle, yak, buffalo, goats, and sheep; but was somewhat different from pig, rat, mouse, human, and chicken versions (Figure 1A). MEGA created the phylogenetic tree of KAT2B proteins under neighbor-joining analysis. As shown in Figure 1B,C, cattle, buffalo, yak, goats, and sheep were clustered together; and the humans, pigs, mice, rats, and chickens are away from the bovine. Seven significant motifs of the KAT2B proteins were found among the 11 species by MEME, and the motifs occupied the exact locations (Figure S2). By searching for KAT2B protein structures through NCBI CDD, three specific conserved domains, including homology (Cdd: pfam06466), NAT (N-Acyltransferase) superfamily (Cdd: cd04301), and bromodomain (Cdd: cd05509), were found in all 11 species (Figure S3). These findings suggest that the KAT2B protein is highly conserved and may play key roles in animals.

### 3.2. Bovine KAT2B Gene Expression Profile

**Figure 2:**
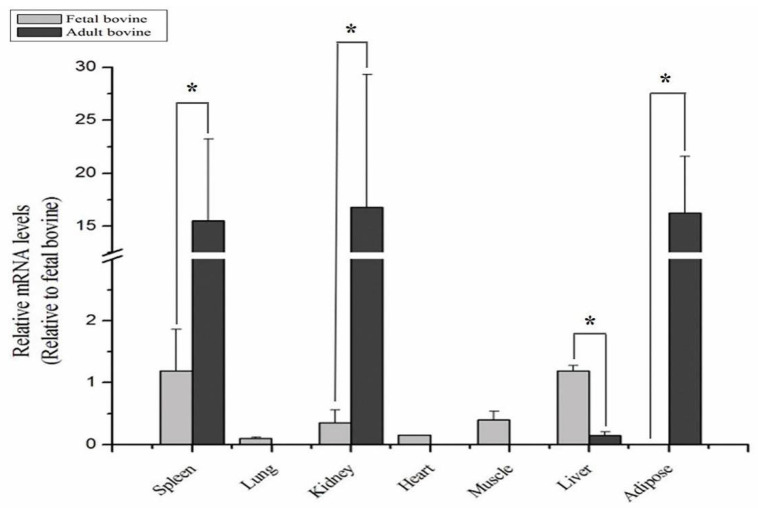
The *KAT2B* gene mRNA expression profile in Qinchuan cattle. * Indicates a significant difference between two groups (*p* < 0.05). Heart in the fetal bovines was the control tissue.

To verify whether *KAT2B* regulates cattle growth and development, we detected *KAT2B* mRNA expression in seven fetal and adult bovine tissues (spleen, lung, kidney, heart, muscle, liver, and adipose). In fetal bovines, the highest *KAT2B* expression level was found in the liver, followed by the spleen and muscle, and the lowest was found in the adipose. In adult bovines, the highest *KAT2B* expression level was found in the kidney, followed by the adipose and spleen. The *KAT2B* mRNA was dramatically increased in the adult bovine spleen, kidney, and adipose, whereas it was almost absent in the adult bovine heart and muscle tissues (Figure 2). These findings suggest that the *KAT2B* gene expression has spatiotemporal specificity during fetal and adult periods, particularly in tissues (e.g., liver, adipose, and muscle) that are closely related to economic traits.

### 3.3. Identification of KAT2B SNPs

**Figure 3:**
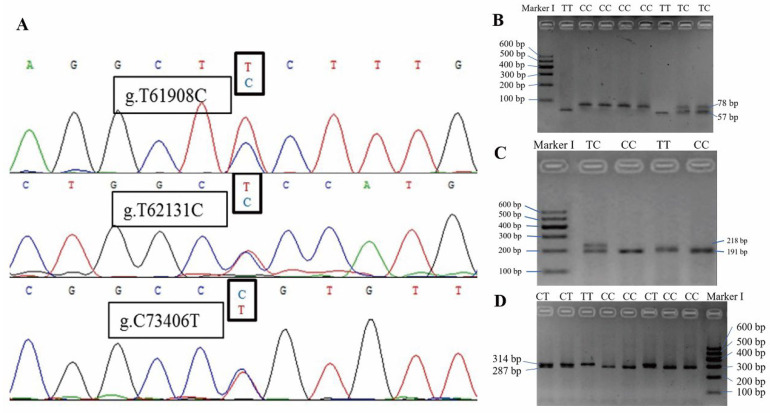
Sequence variants of the *KAT2B* gene in bovines. (**A**) *KAT2B* gene polymorphism sites. (**B**) Genotyping of g.T61908C. TT genotype is represented by the combination of 21 and 57 bp bands; CC genotype is represented by 78 bp band; TC genotype is represented by the combination of 78, 57, and 21 bp bands. (**C**) Genotyping of g.T62131C. The combination of 191 and 27 bp bands represents the CC genotype; the 218 bp band represents the TT genotype; the combination of 218, 191, and 27 bp bands represents the TC genotype. (**D**) Genotyping of g.C73406T. The combination of 287 and 27 bp bands represents the CC genotype; the 314 bp band represents the TT genotype; the CT genotype is represented by the combination of 314, 287, and 27 bp bands.

To find potential molecular markers in the *KAT2B* gene for improvement in cattle breeding, we firstly detected the variations of the *KAT2B* gene using the DNA pooling sequencing method. Three polymorphic sites were detected, including g.T61908C (SNP1), g.T62131C (SNP2), and g.C73406T (SNP3) (Figure 3). Then, the genotype frequency and allele frequency of the three SNPs of *KAT2B* genes in four cattle breeds were calculated (Table 2). SNP1 had mid-genetic diversity in Qinchuan, Chaidam, Yak, and Fu cattle (0.25 < *PIC* < 0.50). SNP2 showed low genetic diversity in Yak cattle (*PIC* < 0.25) and mid-genetic diversity in the other three cattle breeds (0.25 < *PIC* < 0.50). SNP3 had mid-genetic diversity in all four cattle breeds (0.25 < *PIC* < 0.50). Furthermore, SNP1 and SNP3 were within the Hardy–Weinberg equilibrium in all four cattle breeds (*p* > 0.05). SNP2 was within the Hardy–Weinberg equilibrium for all but Yak.

### 3.4. Correlation Analysis of Bovine KAT2B Gene SNPs and Body Measure Traits

The relationships between the *KAT2B* SNPs and the body measurements among four Chinese native cattle breeds were investigated. The polymorphisms of g.T61908C and g.T62131C were significantly associated with body length and circumference of cannon bone in Fu cattle, and TT was the superior genotype. g.T62131C polymorphism was significantly associated with body height, chest width, hip width, and wither height in Qinchuan cattle; and TC was the superior genotype. The g.C73406T polymorphism was significantly associated with body mass; and TT and TC were superior genotypes in Yak and Chaidam cattle, respectively. In addition, g.C73406T was also associated with chest girth and circumference of the cannon bone in Chaidam cattle; and the CC was the superior genotype (Table 3).

## 4. Discussion

The growth and slaughter characteristics of livestock are important indexes for assessing their meat production and economic value. Early selection of individuals with superior phenotypes based on genotype will greatly improve the efficiency of the population. This study analyzed the protein structure and expression profile of *KAT2B*, and the associations of *KAT2B* polymorphisms with body measure traits in 827 bovine individuals. The spatiotemporal-specific expression profiles and three SNPs of the *KAT2B* gene were identified. We found that these SNPs were correlated with multiple body measurements in Fu, Qinchuan, Yak, and Chaidam cattle. This provides a potential molecular marker for improving Chinese cattle and may spark further interest in the underlying mechanism of the *KAT2B* gene’s regulation on bovine growth and development.

KAT2B is an acetyltransferase that belongs to the GNAT family, one of the three prominent acetyltransferases [26]. The KAT2B protein has three conserved functional domains: the homology domain, AT domain, and bromodomain [26]. The homology domain is located in the N-terminal domain. The AT domain and bromodomain are located in the C-terminal domain [27]. Meanwhile, the central region of the AT domain mediates acetyl CoA binding and catalysis. The areas at both ends mediate histone substrate specificity [28]. The KAT2B protein function needs the combined actions of homology and AT domains [29]. It can affect transcription initiation by modifying the position of the chromatin domain, and consequently playing an essential role in cell proliferation [30]. This work found that KAT2B protein motifs and structure were highly conserved among bovines, implicating that the gene can be inherited stably and functions similarly in bovines; it has crucial roles. The *KAT2B* gene expression profile in Qinchuan cattle revealed the spatiotemporal specificity for bovine adipose tissue. This is consistent with our previous observation by the RNA-seq method, which also detected markedly different expression in the adipose tissue of Qinchuan cattle in a different period [14]. The high mRNA expression level of *KAT2B* in the adult period indicates its key role in bovine fat deposition. Teruo Yamauchi et al. [31] found that *KAT2B* was expressed early in E12.5 embryos and played an important role in mouse embryo development [31]. A study found that *KAT2B* can affect the growth and development of murine skeletal muscle by affecting the β-catenin signaling pathway in myoblasts [32]. Tushar K. et al. [33] reported that *KAT2B* regulated heart and forelimb development by acetylating TBX5. According to Rabhi et al. [34], mice’s germline and β cell-specific disruption of the *KAT2B* gene led to impaired insulin secretion and glucose intolerance. Taking these findings together, *KAT2B* is possible to be a candidate gene regulating the development and metabolism of muscle and adipose, and the genetic effects may vary from species and developmental period.

The body measurements are the key indicators of beef cattle’s economic value. Body measure traits, such as body mass and body length, can be utilized to assess carcass meat production. Multiple SNPs in various genes have been demonstrated to be associated with body measurement and meat quality traits [8]. Identification of a gene’s causal variant can promote the accuracy of genomic selection, which is considered as an efficient way of analyzing the associations between genetic polymorphisms and traits of economic importance [4,35]. This study identified three SNPs of the *KAT2B* gene that were associated with different body measure traits among cattle breeds but varied within breeds. These results might result from the differences in the genetic backgrounds of different breeds. Body measure traits are quantitative traits that are usually controlled by multiple genes and their interactions [36]. A candidate gene may have striking effects in one breed while having quite limited effects in another breed due to the negative effects of other genes or epistatic interaction of the candidate gene with other genes [36]. For example, Zhang et al. [37] reported that two indels (14 bp indel and 17 bp indel) in the *SPAG17* gene were related to the Shaanbei white cashmere goat’s body measurement traits but had no significant association with those of Hainan black goat. Liu et al. [38] found that the individuals with copy-number-gain of *SHH* exhibited better performance across breeds, but its effects on body measure traits were different—e.g., chest depth in Qinchuan cattle; body weight and length in Nanyang cattle; and chest girth and body weight in Jinnan cattle. Therefore, breed difference is another factor that should be taken into account in cattle selection breeding. For example, in Fu cattle, individuals with the TT genotype at the g.T61908C locus had better performance in BL, whereas individuals with the CC genotype at the g.T62131C locus had the worst performance in CCB. Hence, in Fu cattle breeding, increasing the TT (g.T61908C) genotype’s frequency or reducing the rate of the CC (g.T62131C) genotype’s frequency in the population may improve growth performance. For Qinchuan cattle, individuals with the TC and TT (g.T62131C) genotypes (respectively) performed better in body height, chest width, hip width, and wither height than those with the CC (g.T62131C) genotype. Therefore, increasing the TC or TT (g.T62131C) genotype frequency in the population may lead to better body measurements in Qinchuan cattle breeding. For Yaks, individuals with the TT (g.C73406T) genotype performed better in body mass. However, for Chaidam cattle, individuals with TT (g.C73406T) performed worst in body mass, chest girth, and circumference of the cannon bone. Therefore, different genotypes should be selected according to their different effects on phenotype.

Meanwhile, we acknowledge that the samples were limited in this study, especially those of Fu, Yak, and Chaidam cattle. Thus, the results need to be verified in more samples. Additionally, further investigation on the mechanisms of *KAT2B*’s influences on bovine body measure traits is necessary for the future.

## 5. Conclusions

This study was the first investigation of the *KAT2B* gene and its SNPs’ effects in four Chinese cattle populations. The analysis of bioinformatic function and expression levels in different bovine tissues suggests important roles of *KAT2B* in cattle. The association analysis revealed the significant effects of three *KAT2B* gene polymorphisms, T61908C, g.T62131C, and g.C73406T, on body measure traits in Qinchuan, Fu, Yak, and Chaidam cattle. These findings suggest that *KAT2B* gene polymorphisms can be used as the molecular markers for early molecular-marker-assisted selection in beef cattle breeding programs.

## Figures and Tables

**Table 1:** Primers for PCR in this study.

Targeted SNP or Gene	Accession Number	Primer Pair	T_m_ (°C)	Name	Position	Sequence (5′-3′)
g.T61908C	AC_000158.1	P1	68	F1	61,883–61,907	GGGTTCCCACTGCACAGGCCAAGCT
				R1	61,936–61,960	GTCCATCAGACGCCCCCACACAGAG
g.T62131C	AC_000158.1	P2	64	F2	62,105–62,129	ACCTTCAAGGCCTTTTACATGCGGG
				R2	62,298–62,322	TCAAAGAGGAATGGACACAGGCAGA
g.C73406T	AC_000158.1	P3	61	F3	73,379–73,403	CTCTTCCCAGTCTCACTTTTGTGGG
				R3	73,668–73,692	AGGCACACTGTTTGATGAGTTTCTA
*KAT2B*	XM_019966941.1	P	60	F	7–26	CGGTCTCTTGACCTTCGTGA
				R	158–177	TTTGCCGGGTATGGAAGGAG
*β*-actin	NM_173979.3	Pr	58	Fr	831–851	GTCATCACCATCGGCAATGAG
				Rr	896–914	AATGCCGCAGGATTCCATG

**Table 2:** Genotype and allele frequencies of SNP of the bovine *KAT2B* gene.

SNP	Breed	Genotype Frequency			Allele Frequency		*χ*^2^ (HWE)	*PIC*	*He*	*Ne*
g.T61908C		CC	TC	TT	C	T				
	Qinchuan (658)	0.13	0.61	0.26	0.43	0.57	*p* > 0.05	0.37	0.49	1.96
	Fu (52)	0.23	0.56	0.21	0.51	0.49	*p* > 0.05	0.37	0.50	2.00
	Yak (48)	0.27	0.58	0.15	0.56	0.44	*p* > 0.05	0.37	0.49	1.97
	Chaidam (69)	0.45	0.35	0.20	0.62	0.38	*p* > 0.05	0.36	0.47	1.89
g.T62131C		CC	TC	TT	C	T				
	Qinchuan (658)	0.39	0.43	0.18	0.61	0.39	*p* > 0.05	0.36	0.48	1.91
	Fu (52)	0.28	0.37	0.35	0.47	0.53	*p* > 0.05	0.37	0.50	1.99
	Yak (48)	0.80	0.10	0.10	0.84	0.16	*p* < 0.01	0.23	0.27	1.37
	Chaidam (69)	0.29	0.38	0.33	0.48	0.52	*p* > 0.05	0.37	0.50	2.00
g.C73406T		CC	CT	TT	C	T				
	Qinchuan (658)	0.47	0.43	0.10	0.69	0.31	*p* > 0.05	0.34	0.43	1.75
	Fu (52)	0.40	0.33	0.27	0.57	0.43	*p* > 0.05	0.37	0.49	1.96
	Yak (48)	0.02	0.44	0.54	0.24	0.76	*p* > 0.05	0.30	0.36	1.57
	Chaidam (69)	0.23	0.55	0.21	0.51	0.49	*p* > 0.05	0.37	0.50	2.00

Note: HWE, Hardy–Weinberg equilibrium; *PIC*, polymorphism information content; He, gene heterozygosity; Ne, effective allele numbers.

**Table 3:** Association analysis of three SNPs of the *KAT2B* gene and body measure traits.

SNP	Breed	Growth Traits	Genotype		
			CC	TC	TT
g.T61908C	Fu	BL(cm)	84.00 ± 7.06 ^a,b^	82.86 ± 8.22 ^b^	89.73 ± 10.04 ^a^
g.T62131C	Fu	CCB(cm)	11.6 ± 1.35 ^A^	10.26 ± 1.49 ^B^	11.39 ± 1.54 ^A^
	Qinchuan	BH(cm)	127.3 ± 6.74 ^b^	130.35 ± 6.07 ^a^	129.26 ± 7.15 ^a,b^
		CW(cm)	36.52 ± 4.75 ^b^	37.91 ± 4.43 ^a,b^	39.48 ± 4.13 ^a^
		HIW(cm)	40.67 ± 5.38 ^b^	43.06 ± 4.78 ^a^	43.5 ± 5.02 ^a^
		WH(cm)	124.31 ± 6.47 ^b^	129.63 ± 10.81 ^a^	127 ± 7.05 ^a,b^
g.C73406T	Yak	BM(cm)	151.00 ± 0.11 ^B^	166.57 ± 9.53 ^B^	177 ± 15.34 ^A^
	Chaidam	BM(cm)	246.36 ± 30.29 ^A,B^	272.27 ± 21.14 ^A^	228.9 ± 57.21 ^B^
		CG(cm)	152.64 ± 7.59 ^A^	141.92 ± 8.12 ^B^	140.2 ± 13.75 ^B^
		CCB(cm)	16.09 ± 1.22 ^A^	14.69 ± 0.95 ^B^	14.3 ± 1.06 ^B^

Note: Values are shown as the least squares means ± standard error. Different superscripts of ^A,B^ and ^a,b^ mean significant differences among groups (*p* < 0.01 or *p* < 0.05). BL = body length, WH = withers height, CCB = circumference of cannon bone, BH = body height, CW = chest width, HIW = hip width, BM = body mass, CG = chest girth.

## Data Availability

Not applicable.

## Supplementary Materials

The following supporting information can be downloaded at: https://www.mdpi.com/article/10.3390/ani12151954/s1, Figure S1: Distribution of phenotypes in Qinchuan cattle; Figure S2: Significant motifs of KAT2B across the 11 species; Figure S3: Structure of KAT2B protein domain families in the 11 species; Table S1: Primers for exploring genetic variations of bovine *KAT2B* gene.
