# Supplementary material for: KAT2B Gene Polymorphisms Are Associated with Body Measure Traits in Four Chinese Cattle Breeds
Source: Animals (Basel). 2022 Aug 1;12(15):1954. doi: 10.3390/ani12151954 (PMC9367347; doi:10.3390/ani12151954)
Supplement: Supplementary file 1 [file animals-12-01954-s001.zip › animals-1769095-supplementary.pdf]

animals

MDPI

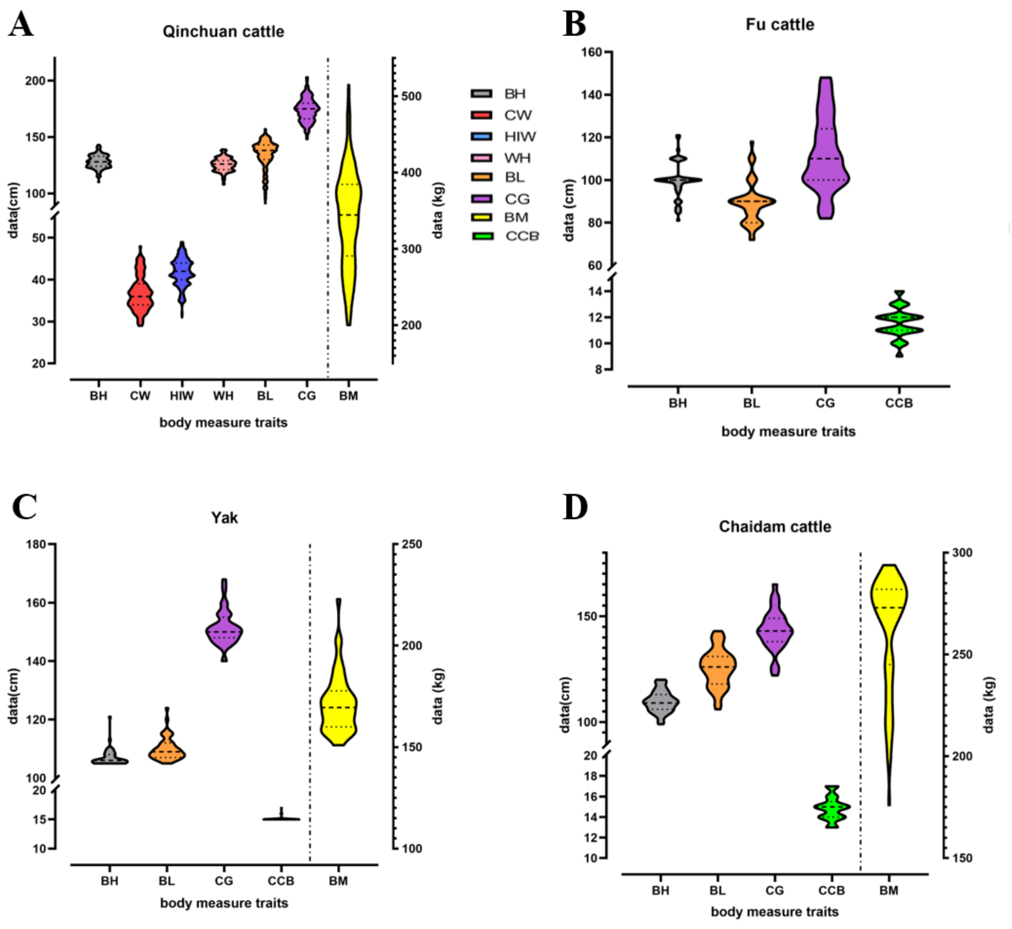

Figure S1. Distribution of phenotypes in Qinchuan cattle. (**A**), Fu cattle (**B**), Yak (**C**), Chaidam cattle (**D**). BH=body height, CW=chest width, HIW=hip-width, WH=withers height, BL=body length, CG=chest girth, CCB=circumference of cannon bone, BM=body mass.

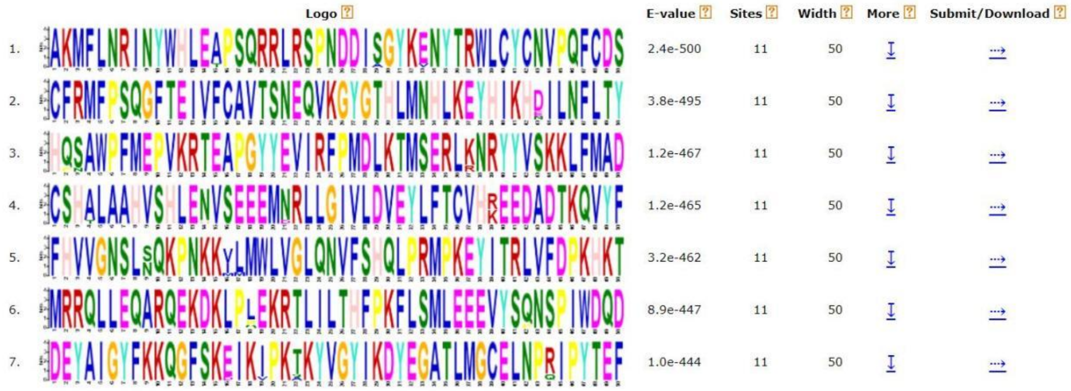

Figure S2. Significant motifs of KAT2B across the 11 species. Motifs were detected using the MEME suite. The different color letters show abbreviations of different amino acids, given through motif analysis on the MEME suite system.

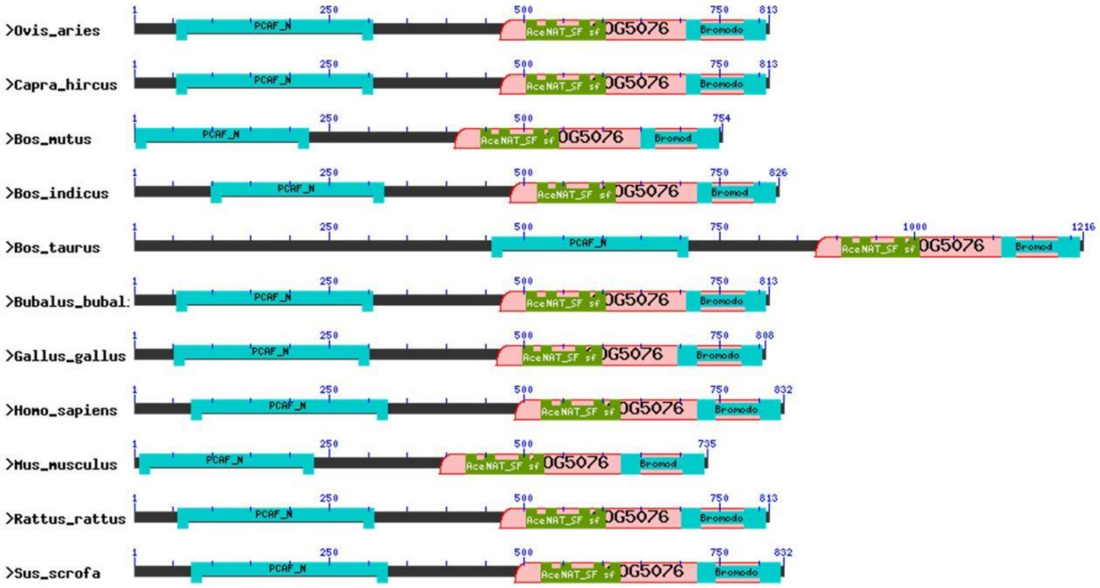

Figure S3. Structure of KAT2B protein domain families in the 11 species. Each color bar is a specific hit representing a different domain superfamily.

Table S1. Primers for exploring genetic variations of bovine *KAT2B* gene.

Targeted Exon	Sequence (5′-3′)	Tm (°C)	Production Size (bp)
Exon1	F: TCCTGGGATGCTCTTGTGCC<br>R: AAGCCATGTTCCTGAGGTGATGTT	63	597
Exon2	F: TCAGCGGAAAAGAAACCAAA<br>R: GGAAAAGTGAGCGGAGACAA	57	827
Exon3	F: TGGCTGTAAGGAAGGGGTGA<br>R: GTGGGTCTTGATGCTCTGTG	55	874
Exon4	F: GTGTCCCATACCTGTCCTCC<br>R: TGTTCTTCACATTGCTCCTG	53	772
Exon5	F: GCAAAGCTGCATGAGTAAAC<br>R: TGGAGATGAGTCTGAGCAAG	52	770
Exon6	F: CTTATCACTTCCTGCGTTTT<br>R: ACCCAAAGTCAACCTCATCT	53	757
Exon7	F: CCTACATGGAGCCCAAAGAG<br>R: GCACTGCACGTCCTGAGACT	57	881
Exon8	F: TTGCTTCCTTATTTCTTGCC<br>R: AACTGCGTTTCTTCCTGCTC	55	796
Exon9	F: AGTCCCTGCTCAAGGAAATC	55	508
Exon10	R: AGCATCAGACAACACGCAAA	56	661
Exon11	F: ACTGCTGTGGACTGTCCCTT	55	706
Exon12	R: ACCCGAGTGGTTCCTCTTTC	54	817
Exon13	F: CCCAAACAGGAGGTGAGACA	54	635
Exon14	R: CCAAAAGACCACATTCCAAA	55	541
Exon15	F: GGAAGGATTGCTGATTTGTT	59	828
Exon16	R: CCCCGTTTTAGAATGTTAGG	55	578
Exon17	F: CAGGATAGTTGGAAAGGAGC<br>R: GTGGGTGAAGGATGAAGACA<br>F: CCGAGATAACTGGCACTACG<br>R: AGCAGAGCAGACGCAAAGGA	48	712

F: TGCGTTACAAGCAGGGTCTG
R: TTGCGTGAAGTGGAGGAAAA
F: GGCTGGAGGATATTGATGGA
R: ACACGCCCTAACCTATGACA
F: GTTTCCCACTTTAGTTCTGT
R: CATTATGTCCAATTCTTTGT
